# Epidemiology of Paediatric constipation in Indonesia and its association with exposure to stressful life events

**DOI:** 10.1186/s12876-018-0873-0

**Published:** 2018-10-03

**Authors:** Hanifah Oswari, Fatima Safira Alatas, Badriul Hegar, William Cheng, Arnesya Pramadyani, Marc Alexander Benninga, Shaman Rajindrajith

**Affiliations:** 1 Department of Child Health, Gastrohepatology Division, Cipto Mangunkusumo Hospital, Faculty of Medicine, Universitas Indonesia, Jakarta, Indonesia; 2 Department of Pediatric Gastroenterology and Nutrition, Emma Children’s, Hospital, Academic Medical Centre, Amsterdam, The Netherlands; 3 Department of Paediatrics, University of Kelaniya, Ragama, 11010 Sri Lanka

**Keywords:** Functional constipation, Prevalence, Risk factor, Symptom, Children, Adolescent

## Abstract

**Background:**

We aimed to study the epidemiology and risk factors, including exposure to emotional stress, for constipation in Indonesian children and adolescents of 10–17 year age group.

**Methods:**

A cross-sectional survey using a validated, self-administered questionnaire was conducted in randomly selected children and adolescents in nine state junior high schools from five districts of Jakarta. All of them were from urban areas. Constipation was defined as a diagnosis by using the Rome III criteria.

**Results:**

Of 1796 children included in the analysis, 328 (18.3%; 95% CI 016–0.2) had constipation. Females and those residing in North Jakarta showed risks associated with constipation in school-age children and adolescents. Symptoms independently associated with constipation were abdominal pain (64% vs 43.3% of control) and straining (22.9% vs 6.3%). The prevalence of constipation was significantly higher in those with stressful life events such as father’s alcoholism (adjusted OR 1.91, 95% CI 1.27–2.89, *P* = 0.002), severe illness of a close family member (adjusted OR 1.77, 95% CI 1.12–2.80, *P* = 0.014), hospitalization of the child for another illness (adjusted OR 1.68, 95% CI 1.22–2.31, *P* < 0.001), being bullied at school (adjusted OR 1.67, 95% CI 1.01–2.76, *P* = 0.047) and loss of a parent’s job (adjusted OR 1.39, 95% CI 1.03–1.88, *P* = 0.034).

**Conclusions:**

Constipation in children and adolescent is a significant health problem, affecting almost 20% of Indonesian school-age children and adolescents. Common school and home related stressful life events appear to have predisposed these children to develop constipation.

## Background

Childhood constipation is considered to be an emerging global public health problem [1]. The prevalence varies among countries from 0.7 to 29.6% [2, 3]. It is estimated that approximately one third of children with constipation suffer from psychological maladjustment [4]. In addition, childhood constipation leads to significant healthcare costs and poor health-related quality of life [5–8]. The aetiology of constipation in children and young adults is not clear. Studies suggest that psychological stress, childhood maltreatment and abnormal childhood personality traits such as hostility, aggression, and negative self-esteem, are associated with functional constipation in children [4, 9, 10]. Some studies have evaluated potential socio-demographic factors, including social class and living area (urban/rural), that may play a role in the development of constipation in children [10–12].

A few studies in Asia have shown that the prevalence of childhood constipation vary between 0.3% in Japan to 32.2% in Taiwan [11–13]. However, there is still a dearth of studies and information to fully understand the clinical epidemiology of this important health problem in Asia [11]. Indonesia has a multi-cultural, multi-lingual and multi-ethnic society. Population of Indonesia is estimated to be over 261 million people. It is the world’s 4th most populous country and the most populous country in the Austronesian region. About 53% of the population live in urban areas. Indonesia has 17,504 islands with over 300 different native languages [14].

We hypothesized that studying the socio-demographic factors, stressful events and bowel habits of the Indonesian children and adolescents would contribute towards the provision of a novel insight into the epidemiology and precipitating factors of constipation in children and adolescents. Therefore, we aimed to study epidemiology, and risk factors, including exposure to emotional stress, for constipation in Indonesian children and adolescents of the 10–17 year age group.

## Methods

### Study design and setting

A cross-sectional study was conducted in all five administrative cities (Municipalities) of Jakarta, Indonesia. Jakarta is the capital of the country and all the selected cities are therefore classified as urban. The inhabitants of these areas are predominantly regular employees (67.2%) and self-employed workers (19.8%) [15].

This study was conducted in 9 state junior high schools in these five administrative cities districts. Data were collected from July 2016 to December 2016 after obtaining consent from School Principals of each school. Informed written consent was obtained from the parents and assent was obtained from children and adolescents before commencement of the study.

This data was secured through 2 collection methods: take-home test (July to August 2016) and examination setting (November–December 2016). In the first method, participants filled the questionnaires at home while in the second, children were asked to fill the questionnaires at school in the presence of a research assistant and in a virtual examination setting. Sensitivity analysis was carried out to identify differences, if any, regarding the collection methods.

### Participants

Nine schools from five districts of Jakarta were selected for this study with convenient sampling and requiring each district to have at least one school. Study participants were gathered consecutively in each school with inclusion criteria: age 10 to 16 years of age and generally in a good health condition. Data included into this study was obtained using the self-administered Questionnaire on Paediatric Gastrointestinal Symptoms which was modified from that used in previous studies [11].

To be eligible to be in the study, participants data regarding subjects’ bowel habits and defecation behaviour, demographic data, family characteristics and other symptoms should have been completed. This study was approved by the Medical Ethics Committee of the Universitas Indonesia.

### Study size

A previous study about constipation in adolescent population conducted in Sri Lanka had reported a prevalence of 15.3% [11]. Since Indonesia and Sri Lanka have some similarities in diet and socio-demographic factors, an anticipated prevalence rate of 15% with 80% of power, 5% significance and 20% of attrition were used for the sample size collection. Based on the above, the minimum sample size for each study site was calculated to be 245.

### Variables

The Rome III criteria were used to define constipation [16]. They include,

1. Defecation frequency of less than three stools/week
2. At least one episode of faecal incontinence
3. A history of retentive posturing or excessive stool retention
4. A history of painful or hard bowel movements
5. A history of a large diameter stool that may obstruct the toilet

Children were considered to have constipation, if they fulfilled at least 2 of the above 5 criteria for at least 2 months. Although the Rome criteria state the presence of palpable faecal mass in the abdomen or rectum as a criterion, we could not practically use this in our epidemiological survey as it was not possible to conduct a physical examination in the setup that was used. Rectal examination was not approved because of limited examination facilities and for ethical reasons.

Stress events included in this study, with reference to the previous study from Sri Lanka were: change in school, suspension from school, frequent punishment in school, separation from the best friend, sitting for a national school examination, failure in school examinations, being bullied at school, severe illness in a close family member, death of a close family member, loss of a parent’s job, divorce or separation of parents, remarriage of divorced parents, birth of a sibling, frequent domestic fights, frequent punishment by the parents, father’s alcoholism, hospitalization of the child for another illness, and exposure to at least 1 stressful event. Each stress event was asked for in the questionnaires given and then separately analysed for association with constipation.

### Data analysis

Data were analysed using SPSS version 23 (IBM Corp. Armonk, NY, USA). *P* < 0.05 was considered statistically significant and 95% confidence interval (CI) was calculated to describe the odd ratio for each variable. Multiple logistic regression analysis was performed on socio-demographic factors and stressful life events that were found to have significant association with constipation during univariate analysis (Chi-squared test or Fisher’s exact test). The 95% CI was measured and attuned to obtain the adjusted odd ratio.

## Results

Three thousand five hundred and seventy-two (3572) questionnaires were distributed in total through the first and second methods listed in the Methods. In the first round (July to August 2016), 2718 questionnaires were distributed and only 1196 (44%), were returned. Then out of that, 1007 (37.1%) questionnaires had all the necessary data to be included into the analysis. In the second round (November–December 2016), 854 questionnaires were distributed, and children were asked to fill the questionnaires at school in the presence of a research assistant. From that, 811 (95%) questionnaires were collected, and 789(92.4%) questionnaires had complete data to be included into the analysis. Therefore, 2007 questionnaires were collected, of which 1796 (89.5%) were included in the analysis; males 732 (40.8%), mean age 13.58 years, SD 0.992 years. Two hundred and eleven (211) questionnaires were excluded due to incomplete socio-demographic details and bowel habit descriptions that would affect the result. In the context that participants recruited by the second method had higher proportion of the return rate compared to the first method, it is possible that this might have resulted in some bias. Therefore, sensitivity analysis was carried out and that showed that there was no difference in the results for the two methods.

### Epidemiology and risk factors

Three hundred and twenty-eight children and adolescents (18.3%; 95% CI 0.16–0.2) met the Rome III criteria for constipation. Table 1 shows the distribution of subjects according to socio-demographic characteristics and prevalence of constipation in each category. Following multivariate analysis, only female gender (adjusted odd ratio 1.319, 95%CI 1.018–1.709, *p* = 0.036) and those residing in North Jakarta (adjusted odd ratio 1.832, 95%CI 1.199–2.799, *p* = 0.005) showed a higher risk of developing constipation .

**Table 1:** Distribution of subjects according to sociodemographic characteristics and prevalence of constipation in each category

Variable		Distribution of subjects		Prevalence of constipation in each category (%)	Crude OR (CI 95%)	*p* value	Adjusted OR (95%CI)	Adjusted *p* value
		Controls *N* = 1468, n(%)	Children with constipation *N* = 328, n(%)					
Age	10–12 y.o	186 (12.7)	31 (9.5)	14.3	1.00 (ref)	–	1.00 (ref.)	–
	13–15 y.o	1268 (86.4)	292 (89.0)	18.7	1.382 (0.925–2.063)	0.114	1.466 (0.944–2.276)	0.089
	16–17 y.o	14 (1)	5 (1.5)	26.3	2.143 (0.721–6.371)	0.17	2.229 (0.727–6.834)	0.161
					*p* = 0.188			
Sex	Male	617 (42)	115 (35.1)	15.7	1.00 (ref)	–	1.00 (ref.)	–
	Female	851 (58)	213 (64.9)	20	1.343 (1.047–1.723)	0.02	1.319 (1.018–1.709)	0.036
					p = 0.02			
Family size	Only child	116 (7.9)	18 (5.5)	13.4	1.00 (ref)	–	1.00 (ref.)	–
	2–3 children	1083 (73.8)	235 (71.6)	17.8	1.398 (0.835–2.343)	0.203	4.736 × 10^8 (0)	1
	≥4 children	269 (18.3)	75 (22.9)	21.8	1.797 (1.028–3.141)	0.04	6.282 × 10^8 (0)	1
					*p* = 0.076			
Birth order^a^	Eldest	509 (37.6)	122 (39.4)	19.3	1.00 (ref)	–	1.00 (ref.)	–
	Youngest	490 (36.2)	104 (33.5)	17.5	0.886 (0.663–1.183)	0.41	0.854 (0.634–1.151)	0.3
	Other	354 (26.2)	84 (27.1)	19.2	0.990 (0.727–1.349)	0.949	0.867 (0.613–1.226)	0.419
					*p* = 0.675			
Mother^b^	Housewife	1058 (72.3)	246 (75.0)	18.9	1.00 (ref)	–	1.00 (ref.)	–
	Employed	405 (27.7)	82 (25.0)	16.8	0.871 (0.661–1.146)	0.324	1.880 (0.853–4.145)	0.117
					*p* = 0.324			
Father’s employment	Leading profession (eg. Doctor, engineer)	16 (1.1)	4 (1.2)	20	1.00 (ref)	–	1.00 (ref.)	–
	Lesser profession (eg. Nurse, teacher)	451 (30.7)	100 (30.5)	18.1	0.887 (0.290–2.710)	0.833	0.790 (0.252–2.477)	0.686
	Skilled non-manual (eg. Clerk)	653 (44.5)	132 (40.2)	16.8	0.809 (0.266–2.457)	0.708	0.767 (0.247–2.382	0.646
	Skilled manual	207 (14.1)	56 (17.1)	21.3	1.082 (0.348–3.366)	0.892	0.901 (0.282–2.880)	0.861
	Unskilled/unemployed	141 (9.6)	36 (11)	20.3	1.021 (0.322–3.242)	0.972	0.886 (0.267–2.939)	0.843
					*p* = 0.513			
Maternal employment	Leading profession (eg. Doctor, engineer)	12 (0.8)	3 (0.9)	20	1.00 (ref)	–	1.00 (ref.)	–
	Lesser profession (eg. Nurse, teacher)	168 (11.4)	36 (11)	17.6	0.857 (0.230–3.194)	0.818	0.682 (0.176–2.635)	0.578
	Skilled non-manual (eg. Clerk)	179 (12.2)	28 (8.5)	13.5	0.626 (0.166–2.357)	0.488	0.539 (0.137–2.125)	0.377
	Skilled manual	16 (1.1)	2 (0.6)	11.1	0.500 (0.072–3.477)	0.484	0.32 (0.044–2.354)	0.263
	Unskilled/unemployed	1093 (74.5)	259 (79.0)	19.2	0.948 (0.266–3.383)	0.934	1.332 (0.293–6.059)	0.711
					*p* = 0.339			
Location of school	South Jakarta	370 (25.2)	67 (20.4)	15.3	1.00 (ref)	–	1.00 (ref.)	–
	North Jakarta	162 (11)	58 (17.7)	26.4	1.977 (1.329–2.941)	0.001	1.832 (1.199–2.799)	0.005
	Central Jakarta	264 (18.0)	73 (22.3)	21.7	1.527 (1.058–2.205)	0.024	1.469 (0.989–2.181)	0.057
	East Jakarta	349 (23.8)	70 (21.3)	16.7	1.108 (0.769–1.596)	0.584	0.959 (0.650–1.413)	0.831
	West Jakarta	323 (22)	60 (18.3)	15.7	1.026 (0.702–1.499)	0.895	0.984 (0.662–1.465)	0.938
					*p* = 0.002			

^a^Family with more than one child

^b^Living mother

### Bowel habits of children with constipation

Bowel habits of children with constipation are depicted in Table 2. Withholding behaviour was present in 68.3% of subjects with constipation. Defecation less than 3 times per week was found in 64.6% of subjects. Presence of hard stools was seen in 63.4%.

**Table 2:** Bowel habits of children with constipation

Bowel habits	Category	n (Percentage)
Defecation frequency	< 3 per week	212 (64.6)
	3–6 per week	24 (7.3)
	Once daily	56 (17.1)
	> One per day	36 (11.0)
Stool Consistency	Hard	206 (63.4)
	Normal	107 (32.9)
	Soft	15 (4.5)
Large diameter stools	Yes	88 (26.8)
	No	240 (73.2)
Withholding posture	Yes	224 (68.3)
	No	104 (31.7)
Painful bowel motions	Yes	180 (54.9)
	No	148 (45.1)
Fecal incontinence	Yes	100 (30.6)
	No	228 (69.4)

Concerning the most common other bowel related symptoms associated with constipation, Table 3 shows that abdominal pain (OR 2.33, 95% CI 1.82–2.98; *P* < 0.001), loss of appetite (OR 1.51 95% CI 1.16–1.97; *P* = 0.002) and straining during defecation (OR 3.87, 95% CI 3.14–6.11; *P* < 0.001) were associated with constipation. However, following multiple logistic regression analysis, only abdominal pain (adjusted OR 2.14, CI 1.66 to 2.77 *P* < 0.001) and straining (adjusted OR 3.87, CI 2.744 to 5.446; *P* < 0.001) were found to be independently associated with constipation.

**Table 3:** Symptoms associated to constipation

Symptoms	Groups		Constipation versus controls	
	Constipation, n(%)	Controls, n(%)	OR (95% CI)	*p* value^*^
Abdominal pain	210 (64)	636 (43.3)	2.33 (1.82–2.98)	< 0.0001
Nausea	1 (0.3)	9 (0.6)	0.49 (0.06–3.93)	0.7^**^
Vomiting	14 (4.3)	36 (2.5)	1.77 (0.95–3.33)	0.07
Loss of appetite	97 (29.7)	320 (21.8)	1.51 (1.16–1.97)	0.002
Straining	75 (22.9)	93 (6.3)	4.38 (3.14–6.11)	< 0.0001

^*^Chi-squared test and ^**^Fisher’s Exact test

### Association between constipation and stressful life events

Table 4 depicts the association between constipation and exposure to stressful life events. Although several stressful events were significantly associated with constipation during the univariate analysis, following multivariate analysis, father’s alcoholism showed the strongest risk associated with constipation in school aged children (adjusted OR 1.91, 95% CI 1.27–2.89, *P* = 0.002), severe illness of a close family member (adjusted OR 1.77, 95% CI 1.12–2.80, *P* = 0.014), hospitalization of the child for another illness (adjusted OR 1.68, 95% CI 1.22–2.31, *P* < 0.001), being bullied at school (adjusted OR 1.67, 95% CI 1.01–2.76, *P* = 0.047) and loss of parent’s job (adjusted OR 1.39, 95% CI 1.03–1.88, *P* = 0.034) also showed a significant association with constipation.

**Table 4:** Distribution of respondent according to exposure to stressful life events

Stressful event	Constipation n(%)	Controls n(%)	OR (95% CI)	*P* Value^*^
Change in school	38 (11.6)	107 (7.3)	1.67 (1.13–2.47)	0.01
Suspension from school	42 (12.8)	145 (9.9)	1.34 (0.93–1.94)	0.114
Frequent punishment in school	22 (15.4)	63 (4.3)	1.62 (0.98–2.67)	0.058
Separation from best friend	21 (6.5)	66 (4.5)	1.46 (0.88–2.43)	0.138
Sitting for a national school examination	12 (3.7)	42 (2.9)	1.3 (0.68–2.49)	0.43
Failure in school examination	15 (4.6)	44 (3)	1.56 (0.86–2.85)	0.14
Being bullied at school	39 (12)	78 (5.3)	2.43 (1.62–3.64)	< 0.0001
Severe illness in a close family member	46 (14.2)	91 (6.2)	2.49 (1.71–3.63)	< 0.0001
Death of a close family member	29 (11.9)	89 (9.1)	1.36 (0.87–2.11)	0.179
Loss of a parent’s job	180 (55)	606 (41.3)	1.74 (1.36–2.21)	< 0.0001
Divorce or separation of parents	106 (32.4)	264 (20.6)	2.18 (1.67–2.85)	< 0.0001
Remarriage of divorced parents	38 (11.6)	142 (9.7)	1.23 (0.84–1.79)	0.293
Birth of a sibling	3 (0.9)	9 (0.6)	1.51 (0.41–5.59)	0.465^**^
Frequent domestic fights	185 (56.6)	622 (42.4)	1.77 (1.39–2.25)	< 0.0001
Frequent punishment by the parents	130 (39.8)	457 (31.2)	1.46 (1.14–1.87)	0.003
Father’s alcoholism	52 (15.9)	108 (7.4)	2.38 (1.66–3.39)	< 0.0001
Hospitalization of the child for another illness	124 (37.9)	332 (22.6)	2.09 (1.62–2.69)	< 0.0001
Exposure to at least 1 stressful event	82 (25.1)	285 (19.4)	1.39 (1.05–1.84)	0.022

^*^Chi-squared test and ^**^Fisher exact test

## Discussion

This large epidemiological survey describes the prevalence and risk factors for constipation of children and adolescents in Jakarta, Indonesia [17]. Nearly one fifth (18.3%) of Indonesian school children and adolescents aged 10–17 years fulfil the Rome III criteria for constipation. Constipation was more common in girls and adolescents who were exposed to both home and school related stressful life events had higher odds of developing constipation.

The prevalence rate of constipation in Indonesia (18.3%) is slightly higher than most of the studies from the Western world, [US (12.9%) [18], Greece (13.9%) [19]], South-East Asia, [Sri Lanka (15.3%) [11]] and South America, [Mexico (12.6%) [20], and Panama (15.9%) [21]]. In contrast, the prevalence rate of constipation in Taiwan (32.2%) [12] was nearly twice as high as in the current study. However, the prevalence of constipation in Indonesia was markedly higher than China where the prevalence rates range from 3.1 to 12.2% [22–25]. The reason for these differences are not entirely clear. It may have been due to many reasons such as differences in sample selection (community samples vs. school samples), differences in methods of data collection (parental vs. child questionnaires), differences in data collection instruments including subtle alterations in translations, differences in cultural and regional interpretation of bowel habits and other GI symptoms together with differences in diet and behavioural patterns. In addition, there could be a true difference in genetic potential in developing constipation in these populations.

Predominance of a gender in constipation is still far from conclusive. Studies from Panama and Sri Lanka reported no difference between males and females [11, 21]. Whereas Lewis et al. found constipation more prevalent in males than females in the US [18]. In contrast to the latter study we found a higher prevalence in females. Similar to our findings, Wu et al noted a significantly higher prevalence of constipation in females in Taiwan [12]. Adult studies have also shown that constipation was more common in females than males [26]. When we looked further into our data, the predominance of constipation in females might be related to more stressful events in females than males in our study (data not shown). Socio-demographic factors did not show any significant difference between children with constipation and controls in our study.

Only a few studies describe bowel habits of children with constipation. Compared to children with constipation in Sri Lanka and Iran, children and adolescents included in this study had higher occurrence of infrequent stools (< 3/ per week), and hard stools [11, 27]. They were also noted to have higher frequency of posturing and having more faecal incontinence. The frequency of faecal incontinence was higher than that of Brazilian children with constipation [28]. However, pain while passing stools and large diameter stools were less frequent in Indonesian children with constipation compared to Sri Lankan and Iranian children [11, 27]. Of the other symptoms studied, abdominal pain was the only symptom that was independently associated with constipation. A similar observation was noted in the study conducted among Sri Lankan adolescents [11].

Many factors could contribute to the development of constipation in children, such as abnormal personality traits [4], stressful life events [9], child maltreatment [10], dietary habits [29] and obesity [30]. Pressure at home and school could transform into stressful events that lead to constipation. In our study, father’s alcoholism, severe illness in a close family member, hospitalization of the child for another illness, being bullied at school and loss of a parent’s job were clearly associated with constipation. Similarly, another study reported the association between stress and constipation [9]. In that study, separation from the best friend, failure in exam, severe illness among family members, and frequent punishments by parents were associated with constipation. Although there are subtle variations in the findings of that study and the current study, it is evident that home and school related stressful events predispose children to develop constipation. In contrast, a study from Nigeria, using the same list of stressful events, did not find a significant association between constipation and stressful life events. A smaller sample size could have contributed to this lack of difference in the Nigerian study. It had been shown that poor quality of interactions due to marital disharmony, verbal/emotional abuse of children etc., between the child and the caregiver before the age of 18 years could lead to the development of functional gastrointestinal disorders [31].

There are several strengths of this study. We included a large number of children in the study which conferred adequate power to our findings. We also used standard Rome III criteria for the diagnosis of constipation and therefore were able to compare our findings with other studies to draw meaningful conclusions. However, as many of the other epidemiological surveys across the world on this topic, we did not conduct a physical examination on these children nor did we investigate them to rule out the possibility of organic disorders. However, most of the studies have failed to find significant organic disorders in children who have fulfilled Rome criteria for functional gastrointestinal disorders [32, 33] and current guidelines also do not recommend investigating children who fulfil standard criteria for constipation [34].

As an observational study, data were obtained using questionnaires which may be subject to information bias, including recall bias. As this study was conducted using two different methods for data collection (questionnaires filled at home and questionnaires filled in the school), sensitivity analysis was performed between the two methods. In sociodemographic factor, gender was found to be not significant in the second method (*p* = 0.378) while the first method showed the same result with the total participants (*p* = 0.027). This could have occurred because of differences in gender proportion (60.7% subjects are girls). We did not find any other significant differences in the sensitivity analysis.

Our findings have noteworthy implications both at national and global levels for clinical and research practices. According to Unicef estimates, Indonesia has around 85 million children, which represents one-third of the national population [17]. Therefore, allocation of healthcare resources for this growing problem of constipation would be an uphill task for policymakers. Our data also provide the understanding of epidemiological distribution of constipation in children at the global level and provide an insight towards predisposing factors. We believe that these findings could contribute to the development of preventive strategies for constipation in children.

## Conclusions

The present study shows that almost 20% of children and adolescents in Indonesia suffer from constipation. This functional gastrointestinal disorder is more common in girls. Some common school and home related stressful live events predispose children to develop constipation and therefore, national, regional and global policymakers need to pay attention to the growing problem of constipation in Indonesian children.

## References

[1] Rajindrajith S, Devanarayana NM, Crispus Perera BJ, Benninga MA (2016). Childhood constipation as an emerging public health problem. World J Gastroenterol.

[2] van den Berg MM, Benninga MA, Di Lorenzo C (2006). Epidemiology of childhood constipation: a systematic review. Am J Gastroenterol.

[3] Mugie SM, Benninga MA, Di Lorenzo C (2011). Epidemiology of constipation in children and adults: a systematic review. Best Pract Res Clin Gastroenterol.

[4] Ranasinghe N, Devanarayana NM, Benninga MA, van Dijk M, Rajindrajith S (2017). Psychological maladjustment and quality of life in adolescents with constipation. Arch Dis Child.

[5] Clarke MC, Chow CS, Chase JW, Gibb S, Hutson JM, Southwell BR (2008). Quality of life in children with slow transit constipation. J Pediatr Surg.

[6] Rajindrajith S, Devanarayana NM, Weerasooriya L, Hathagoda W, Benninga MA (2013). Quality of life and somatic symptoms in children with constipation: a school-based study. J Pediatr.

[7] Youssef NN, Langseder AL, Verga BJ, Mones RL, Rosh JR (2005). Chronic childhood constipation is associated with impaired quality of life: a case-controlled study. J Pediatr Gastroenterol Nutr.

[8] Choung RS, Shah ND, Chitkara D, Branda ME, Van Tilburg MA, Whitehead WE (2011). Direct medical costs of constipation from childhood to early adulthood: a population-based birth cohort study. J Pediatr Gastroenterol Nutr.

[9] Devanarayana NM, Rajindrajith S (2010). Association between constipation and stressful life events in a cohort of Sri Lankan children and adolescents. J Trop Pediatr.

[10] Rajindrajith S, Devanarayana NM, Lakmini C, Subasinghe V, de Silva DG, Benninga MA (2014). Association between child maltreatment and constipation: a school-based survey using Rome III criteria. J Pediatr Gastroenterol Nutr.

[11] Rajindrajith S, Devanarayana NM, Adhikari C, Pannala W, Benninga MA (2012). Constipation in children: an epidemiological study in Sri Lanka using Rome III criteria. Arch Dis Child.

[12] Wu TC, Chen LK, Pan WH, Tang RB, Hwang SJ, Wu L (2011). Constipation in Taiwan elementary school students: a nationwide survey. J Chin Med Assoc.

[13] Sagawa T, Okamura S, Kakizaki S, Zhang Y, Morita K, Mori M (2013). Functional gastrointestinal disorders in adolescents and quality of school life. J Gastroenterol Hepatol.

[14] Wikipedia. Indonesia. https://en.wikipedia.org/wiki/Indonesia#cite_note-IslandPop-14. Accessed 15 Aug 2018.

[15] Statistik BP. Population of 15 years of age and over who are working by region nad employment status of main job-DKI Jakarta Provice. http://sp2010.bps.go.id/index.php/site/tabel?wid=3100000000&tid=270&fi1=58&fi2=3. Accessed 15 Aug 2018.

[16] Waker LS, Caplan A, Rasquin A (2000). Manual for the questionnaire on paediatric gastrointestinal symptoms.

[17] Unicef-Indonesia. The big picture. https://www.unicef.org/indonesia/children.html. Accessed 9 Jan 2017.

[18] Lewis ML, Palsson OS, Whitehead WE, van Tilburg MAL (2016). Prevalence of functional gastrointestinal disorders in children and adolescents. J Pediatr.

[19] Bouzios I, Chouliaras G, Chrousos GP, Roma E, Gemou-Engesaeth V (2017). Functional gastrointestinal disorders in Greek children based on ROME III criteria: identifying the child at risk. Neurogastroenterol Motil.

[20] Dhroove G, Saps M, Garcia-Bueno C, Leyva Jimenez A, Rodriguez-Reynosa LL, Velasco-Benitez CA (2017). Prevalence of functional gastrointestinal disorders in Mexican schoolchildren. Rev Gastroenterol Mex.

[21] Lu PL, Saps M, Chanis RA, Velasco-Benítez CA (2016). The prevalence of functional gastrointestinal disorders in children in Panama: a school-based study. Acta Paediatr.

[22] Tam YH, Li AM, So HK, Shit KY, Pang KK, Wong YS (2012). Socioenvironmental factors associated with constipation in Hong Kong children and Rome III criteria. J Pediatr Gastroenterol Nutr.

[23] Xu HL, Lin SH, Lin LR, Ni MY, Ji XF, Xl W (2008). Epidemiologic survey of children with functional constipation in Jieyang City. Hainan Med J.

[24] Zhang SC, Wang WL, Qu RB, Su PJ, Zhang SW, Zhang HR (2010). et al. Zhonghua Liu Xing Bing Xue Za Zhi.

[25] Zhou HQ, Li DG, Song YY, Zhong CH, Hu Y, Xu XX (2007). An epidemiologic study of functional bowel disorders in adolescents in China. Zhonghua Yi Xue Za Zhi.

[26] Chang L (2004). Review article: epidemiology and quality of life in functional gastrointestinal disorders. Aliment Pharmacol Ther.

[27] Dehghani SM, Kulouee N, Honar N, Imanieh MH, Haghighat M, Javaherizadeh H (2015). Clinical manifestations among children with chronic functional constipation. Middle East J Dig Dis.

[28] De Araujo Sant’Anna AM, Calcado AC (1999). Constipation in school-aged children at public schools in Rio de Janeiro, Brazil. J Pediatr Gastroenterol Nutr.

[29] Kranz S, Brauchla M, Slavin JL, Miller KB (2012). What do we know about dietary fiber intake in children and health? The effects of fiber intake on constipation, obesity, and diabetes in children. Adv Nutr.

[30] Pashankar DS, Loening-Baucke V (2005). Increased prevalence of obesity in children with functional constipation evaluated in an academic medical center. Pediatrics.

[31] Felitti VJ, Anda RF, Nordenberg D, Williamson DF, Spitz AM, Edwards V (1998). Relationship of childhood abuse and household dysfunction to many of the leading causes of death in adults. The Adverse Childhood Experiences (ACE) Study. Am J Prev Med.

[32] Devanarayana NM, de Silva DG, de Silva HJ (2008). Aetiology of recurrent abdominal pain in a cohort of Sri Lankan children. J Paediatr Child Health.

[33] Dhroove G, Chogle A, Saps M (2010). A million-dollar work-up for abdominal pain: is it worth it?. J Pediatr Gastroenterol Nutr.

[34] Tabbers MM, DiLorenzo C, Berger MY, Faure C, Langendam MW, Nurko S (2014). Evaluation and treatment of functional constipation in infants and children: evidence-based recommendations from ESPGHAN and NASPGHAN. J Pediatr Gastroenterol Nutr.

